# Preparation of multifunctional hydrogels with accessible isothiouronium groups via radical cross-linking copolymerization

**DOI:** 10.1038/s41598-023-36956-x

**Published:** 2023-06-26

**Authors:** Jana Grübel, Vanessa L. Albernaz, Anastasia Tsianaka, Corinna O. Jauch, Silia Quirin, Christian Kerger, Christina G. Kohl, Anke Burger-Kentischer, Günter E. M. Tovar, Alexander Southan

**Affiliations:** 1 Institute of Interfacial Process Engineering and Plasma Technology IGVP, University of Stuttgart, Nobelstr. 12, 70569 Stuttgart, Germany; 2 Fraunhofer Institute for Interfacial Engineering and Biotechnology IGB, Nobelstr. 12, 70569 Stuttgart, Germany; 3 Present Address: Max Planck Institute for Intelligent Systems, Heisenbergstr. 3, 70569 Stuttgart, Germany

**Keywords:** Drug delivery, Bioconjugate chemistry, Gels and hydrogels, Organic molecules in materials science, Polymers, Wetting, Polymer synthesis, Gels and hydrogels, Organic molecules in materials science, Polymers, Wetting

## Abstract

Hydrogels can be equipped with functional groups for specific purposes. Isothiouronium groups can enhance adsorptivity, or allow coupling of other functional groups through mild reactions after transformation to thiol groups. Here we present a method to prepare multifunctional hydrogels by introducing isothiouronium groups into poly(ethylene glycol) diacrylate (PEGDA) hydrogels, and convert them into thiol-functionalized hydrogels by the reduction of the isothiouronium groups. For this purpose, the amphiphilic monomer 2-(11-(acryloyloxy)-undecyl)isothiouronium bromide (AUITB), containing an isothiouronium group, was synthesized and copolymerized with PEGDA. In this convenient way, it was possible to incorporate up to 3 wt% AUITB into the hydrogels without changing their equilibrium swelling degree. The successful functionalization was demonstrated by surface analysis of the hydrogels with water contact angle measurements and increased isoelectric points of the hydrogel surfaces from 4.5 to 9.0 due to the presence of the isothiouronium groups. The hydrogels showed a suitability as an adsorbent, as exemplified by the pronounced adsorption of the anionic drug diclofenac. The potential of the functionalization for (bio)conjugation reactions was demonstrated by the reduction of isothiouronium groups to thiols and subsequent immobilization of the functional enzyme horseradish peroxidase on the hydrogels. The results show that fully accessible isothiouronium groups can be introduced into radically cross-linked hydrogels.

## Introduction

Hydrogels are extensively examined in the fields of tissue engineering^1–3^, drug delivery^4–6^ or for (bio)sensors^7–9^. They are composed of an insoluble polymer network that is swollen in an aqueous medium^10^. Hydrogel functionality, like its swelling behavior, generally results from an interplay of polymer network and swelling medium^11–13^. However, an elegant way to tailor hydrogel properties is by changing the polymer network composition or cross-link density and architecture.

One prominent class of hydrogels is based on poly(ethylene glycol) (PEG), often prepared by photocuring of PEG diacrylate (PEGDA)^5,14–16^. The resulting hydrogels are bioinert and inhibit protein adsorption^17,18^ as well as cell adhesion^19,20^. Based on the requirements on the hydrogel properties, the polymer network formation can be influenced by the molar mass or concentration of PEGDA in the hydrogel precursor solutions, *i.e.* before cross-linking^21–23^. Additionally, a functionalization of PEG-based hydrogels can be achieved by adding other molecules or monomers to the hydrogel precursor solutions. This was done successfully before, for example by including monomers generating a polyelectrolyte hydrogel^15,16,24^. The positively charged monomer 2-(methacryloyloxy)ethyl trimethylammonium chloride (MAETAC) was incorporated into PEGDA hydrogels by copolymerization^15^. In a similar study the negatively charged monomer sodium methallyl sulfonate (SMAS) was used to functionalize PEGDA hydrogels^16^. In both studies, increased protein adsorption, cell adhesion, and proliferation were observed on the functionalized hydrogels compared with the non-functionalized hydrogels. Besides enhancing cell compatibility, such polyelectrolyte hydrogels have other advantages and possible applications. Polyelectrolyte materials can be used to adsorb molecules of the opposite charge by electrostatic interaction or ion exchange and can be applied, e.g., in water purification^25^. The choice of the monomer and its functional group is crucial for the possible application field of the hydrogel.

One particularly interesting ionic moiety, which has to our knowledge so far been neglected for the preparation of macroscopic hydrogels, is the isothiouronium group. Remarkable properties of low molar mass isothiouronium salts or isothiouronium-containing nanoparticles, like anti-tumor activity^26^ or anti-bacterial effects^27^, were reported before. Isothiouronium-functional particles are commercially available as adsorbers for heavy metal ions^28–31^. The isothiouronium group is stable in acidic to neutral aqueous environments and in contact with air and might therefore be suitable to be added directly to hydrogel precursor solutions.

Apart from the direct effects of isothiouronium groups, their chemical properties facilitate another possibility: They hydrolyze efficiently to yield thiols under the action of sodium hydroxide or sodium metabisulfite^32,33^. Therefore, isothiouronium groups can be considered as synthetic thiol precursors. This is of particular relevance because it often proves difficult to introduce thiols directly into hydrogels because they interfere with typical cross-linking reactions, e.g. radical polymerization^34,35^ or are a reactant in the cross-linking reaction by which they are consumed, e.g. in polymer cross-linking by thiol-yne or Michael-type addition reactions^36,37^. Thus, the path using isothiouronium groups as protected thiols during cross-linking might offer an elegant opportunity to liberate thiols by post-polymerization reactions after curing.

The high reactivity of thiols offers the opportunity to covalently bind other molecules and even use it for bioconjugation reactions in aqueous environment^38^. However, only a few studies report an availability of thiols in hydrogels. The group of Sajeesh et al. prepared hydrogel micro particles based on polymethacrylic acid, polyethylene glycol and chitosan with a thiol functionality for the oral delivery of insulin^39^. Southan et al*.* synthesized polymers with a PEG backbone and copolymerized poly(glycidylthiol) with PEGDA to generate PEG-based hydrogels with thiol functionality, which increased the cell adhesiveness of the hydrogels to fibroblasts compared to unmodified PEGDA hydrogels^19^. In another study, PEG-based hydrogels were coated with gold nanoparticles via reactive micro-contact printing^40^. Due to the interactions of thiols and gold, the gold nanoparticles were transferred on thiol-functionalized PEG hydrogels more easily and murine fibroblast L929 cells preferred to adhere on the nanoparticle layer. Cai et al*.* prepared PEG hydrogels via nucleophilic thiol-yne addition with 4-arm PEG-alkynes and PEG-thiols^36^. They determined residual thiols in the cross-linked hydrogels, hence there must have been free alkynes. And they used the free alkynes to bind a thiol-containing dye as well as an antimicrobial peptide but did not use the thiols in the hydrogel.

In this paper, we address the hypothesis of whether the newly synthesized isothiouronium-functional group-bearing monomer 2-(11-(acryloyloxy)-undecyl)isothiouronium bromide (AUITB) can be used in the formation of polymer networks by radical cross-linking of PEGDA, and thereby generate isothiouronium-functional hydrogels. We then aim to investigate the surface and bulk properties of the isothiouronium-functional hydrogels that can hypothetically be generated in this process, including testing their usefulness as adsorbents or antimicrobial materials. In addition, we would like to pursue the hypothesis that isothiouronium-functional hydrogels can be used to generate accessible thiol groups in the hydrogels that exhibit reactivity in the thiol-Michael reaction. Finally, we would investigate whether the thiols thus introduced can be used to covalently immobilize an enzyme in the hydrogels while maintaining its activity.

## Experimental section

### Chemicals

Following chemicals were purchased from Merck KGaA (Darmstadt, Germany): 2,2´-azino-bis(3-ethylbenzothiazoline-6-sulfonic acid) (ABTS) liquid substrate solution, ammonium hydroxide (NH_4_OH) ~ 28–30% NH_3_ basis, acryloyl chloride ≥ 97%, deuterated chloroform (CDCl_3_) 99.8%, diiodomethane 99%, ethanol (EtOH) absolute for analysis, hydrogen peroxide (H_2_O_2_) 30%, hydroquinone, 2-hydroxy-4′-(2-hydroxyethoxy)-2-methylpropiophenone (Irgacure 2959), poly(ethylene glycol) diacrylate (PEGDA) *M*_n_ ~ 700 g mol^−1^, sodium hydroxide (NaOH), sodium metabisulfite (Na_2_S_2_O_5_), potassium chloride (KCl) for analysis, triethylamine (TEA) ≥ 99.5%, and trifluoroacetic acid (TFA), for HPLC ≥ 99%. Chloroform ≥ 99.8% and dichloromethane (DCM) ≥ 99.8% were purchased from Honeywell (Offenbach, Germany). Technical acetone and isopropanol were purchased from Brenntag GmbH (Essen, Germany). Dimethyl sulfoxide (DMSO) for synthesis and tetrahydrofuran (THF) for HPLC were purchased from ChemSolute® (Th.Geyer, Renningen, Germany). 11-bromo-1-undecanol and diclofenac sodium salt (DSS) ≥ 98.0% were purchased from TCI GmbH (Eschborn, Germany). Acetonitrile, ROTISOLV® HPLC gradient grade, sodium bicarbonate, sodium chloride and tryptone were purchased from Carl Roth GmbH + Co. KG (Karlsruhe, Germany). Ethylene glycol 99% and thiourea were purchased from Alfa Aesar (Kandel, Germany). EZ-Link™ maleimide-PEG_11_-biotin and yeast extract (Gibco) were purchased from Thermo Fisher Scientific (Dreieich, Germany). Other reagents were purchased from the following companies (given in parentheses): Atto488 maleimide (Atto-TEC GmbH, Siegen, Germany), BD Bacto agar (BD Becton Dickinson GmbH, Heidelberg, Germany), deuterated dimethyl sulfoxide (DMSO-d_6_) 99.8% (Deutero, Kastellaun, Germany), ethyl acetate for HPLC (VWR Chemicals, Darmstadt, Germany), hydrochloric acid (HCl) 37% (Häberle Labortechnik GmbH & Co, Lonsee-Ettlenschieß, Germany), magnesium sulfate 99% anhydrous (abcr GmbH, Karlsruhe, Germany), nitrogen gas (Air Liquide, Düsseldorf, Germany), potassium hydroxide (KOH) (AppliChem GmbH, Darmstadt, Germany), streptavidin-coupled poly horseradish peroxidase (SA-HRP, SDT reagents, Baesweiler, Germany). Phosphate buffered saline (PBS, pH 7.2) was freshly prepared with 137 mM NaCl, 2.7 mM KCl, 1.5 mM KH_2_PO_4_ and 8.1 mM Na_2_HPO_4_ · 2 H_2_O in deionized water. Acryloyl chloride was distilled before use. Lysogeny broth (LB) agar was prepared with 10 g L^−1^ tryptone, 5 g L^−1^ yeast extract, 10 g L^−1^ NaCl and 15 g L^−1^ agar. The pH was adjusted to 7.0 with 1 N NaOH and the LB agar was subsequently sterilized by autoclaving. LB medium was prepared with 10 g L^−1^ tryptone, 5 g L^−1^ yeast extract and 10 g L^−1^ NaCl. The pH was adjusted to 7.0 with 1 N NaOH and the LB medium was subsequently sterilized by autoclaving.

### Synthesis of 11-bromoundecyl acrylate

5.00 g (19.9 mmol, 1.0 eq.) of 11-bromo-1-undecanol were dissolved in 70 mL tetrahydrofuran (THF) and cooled in an ice bath. 3.33 mL (23.9 mmol, 1.2 eq.) triethylamine were added. Under argon atmosphere, 1.93 mL (23.9 mmol, 1.2 eq.) acryloyl chloride were dissolved in 30 mL THF and added dropwise to the flask (approx. 1 drop/3 s). The reaction mixture turned opaque and was stirred at room temperature for 40 h. The mixture was filtrated and THF was removed under reduced pressure at 160 mbar and 25 °C. 50 mL dichloromethane (DCM) were added and the organic phase was extracted with 50 mL of a 2% w/v sodium bicarbonate (NaHCO_3_) solution. The organic phase was dried with magnesium sulfate and the solvent was removed under reduced pressure of 400 mbar at 25 °C. The product (beige to yellow paste) was dried under vacuum for 2.5 h. Other than 11-bromoundecyl acrylate, also 11-hydroxylundecyl acrylate was generated. Since the latter cannot react in the second step, no further purification was performed. 82% of the product mixture (2.85 g) consisted of 11-bromoundecyl acrylate in 38% yield.

^1^H NMR of 11-bromoundecyl acrylate (500 MHz, CDCl_3_): δ [ppm] = 1.35 (m, 14 H), 1.62–1.70 (m, 2 H), 1.80–1.89 (m, 2 H), 3.40 (t, *J* = 6.9 Hz, 2 H), 4.14 (t, *J* = 6.8 Hz, 2 H), 5.81 (dd, *J* = 10.4, 1.4 Hz, 1 H), 6.12 (dd, *J* = 17.3, 10.4 Hz, 1 H), 6.39 (dd, *J* = 17.3, 1.4 Hz, 1 H). The ^1^H NMR spectrum is shown in Figure SI 1.

### Synthesis of 2-(11-(acryloyloxy)undecyl)isothiouronium bromide (AUITB)

2.71 g of the product mixture from the previous reaction, containing 2.22 g (7.3 mmol, 1.0 eq.) 11-bromoundecyl acrylate, were dissolved in 44 mL EtOH. A solution of 0.024 g (0.2 mmol, 0.03 eq.) hydroquinone in 11 mL ethanol and a solution of 0.75 g (9.8 mmol, 1.35 eq.) thiourea in 123 mL ethanol were added. The mixture was stirred under reflux for 6 h. EtOH was removed under reduced pressure at 100 mbar and 40 °C. The mixture was washed with chloroform (CHCl_3_) and filtrated. CHCl_3_ was removed under reduced pressure of 370 mbar at 40 °C, and the residue was dried under vacuum. Ethyl acetate was added to precipitate the product (white solid) and removed by filtration. AUITB was dried under vacuum overnight and was obtained in 36% yield.

^1^H NMR of AUITB (500 MHz, DMSO-d_6_): δ [ppm] = 1.13–1.43 (m, 14 H), 1.50–1.65 (m, 4 H), 3.13 (t, *J* = 7.3 Hz, 2 H), 4.09 (t, *J* = 6.6 Hz, 2 H), 5.93 (dd, *J* = 10.3, 1.6 Hz, 1 H), 6.16 (dd, *J* = 17.3, 10.3 Hz, 1 H), 6.31 (dd, *J* = 17.3, 1.6 Hz, 1 H), 8.99 (s, 4 H).

^13^C NMR of AUITB (126 MHz, DMSO-d_6_): δ [ppm] = 25.35, 27.77, 28.08, 28.34, 28.37, 28.62, 28.83, 28.86, 28.87, 30.11, 64.07, 128.41, 131.36, 165.52, 169.93. The NMR spectra are shown in Figure SI 2 and Figure SI 3.

Elemental analysis (%) calculated for AUITB: C 47.24, H 7.66, N 7.35, S 8.41, Br 20.95, O 8.39; found C 47.37, H 7.69, N 7.43, S 8.34, Br 20.75.

Raman spectra of AUITB: $\nu$ [cm^−1^] = 2846 (C-H), 1714 (*α*,*β*-unsaturated ester), 1637 (NH_2_ or C = C).

### Preparation of functionalized poly(ethylene glycol) diacrylate samples

Poly(ethylene glycol) diacrylate (PEGDA) samples were prepared by radical cross-linking with the photoinitiator Irgacure 2959. For PEGDA samples without AUITB (PEGDA-0), a precursor solution of 0.5 wt% Irgacure 2959 and 99.5 wt% PEGDA was prepared and mixed overnight under the protection of light. For functionalized samples, between 0.1 wt% and 3 wt% AUITB were dissolved in PEGDA and, if needed to facilitate dissolution, additional heating at 80 °C was performed, followed by cooling to room temperature. This resulted in a molar ratio of AUITB and PEGDA between 0.18% and 5.7%. Afterwards, Irgacure 2959 was added to a final concentration of 0.5 wt% and dissolved overnight under the protection of light. The composition of all hydrogel precursor solutions is listed in Table SI 1. AUITB containing samples are referred to as PEGDA-0.1, PEGDA-0.5, PEGDA-1, PEGDA-2 and PEGDA-3 further on, the number meaning the AUITB mass fraction *β*_AUITB_ in the precursor solutions. Silicon wafers were cleaned with acetone, isopropanol and deionized water and dried with nitrogen gas after each cleaning step. Afterwards, the clean wafers were activated with hydrogen peroxide and ammonium hydroxide in a volume ratio of 2:3 at 70 °C for 40 min. After cooling down to room temperature (RT), the wafers were rinsed with deionized water and dried with nitrogen gas. Quartz glass panes were rinsed with acetone and deionized water. The activated silicon wafers were transferred to aluminum molds for better stability and silicone frames (height 500 µm) were placed on top. The PEGDA solution was poured inside the frame and the mold was closed with the quartz glass pane. The solution was cross-linked with UV-light for 7.5 min after a waiting period of 5 h under the protection of light (radiation intensity of 50 mW cm^−2^, spectral range > 300 nm, with an emission maximum around approx. 365 nm, sol2, Dr. Hönle AG). For surface analysis, the hydrogel surface orientated towards the activated silicon wafers was always investigated. For adsorption measurements, precursor solutions were prepared as described before with 0.5 wt% Irgacure 2959, *β*_AUITB_ between 1 wt% and 3 wt%, and between 98.5 wt% and 96.5 wt% PEGDA (Table SI 1). After mixing, the PEGDA solution was poured into a cylindrical aluminum mold (diameter 30 mm, height 1 mm), covered with a quartz glass pane and subsequently cross-linked with UV irradiation for 7.5 min. Before subsequent experiments with the samples, they were washed excessively and swollen as explained in the respective sections below.

### Raman spectroscopy

Raman spectra (inVia™ Raman microscope, Qontor®, Renishaw plc) were recorded of AUITB, non-reacted PEGDA, a PEGDA-0 sample and a PEGDA-3 sample to evaluate if the monomer interfered with the cross-linking process of PEGDA and if it was possible to detect the isothiouronium groups of AUITB. Samples were measured after swelling in water for 72 h and drying under reduced pressure of approx. 50 mbar for 24 h at 60 °C (VDL 53, Binder GmbH). For recording the spectra, a 532 nm laser and the objective lens Leica 50x/0.5/8.2 mm were used. Baseline corrections of the spectra were performed with a self-implemented version of a modified polynomial fitting algorithm according to Lieber & Mahadevan-Jansen in the wavenumber range from 700 to 3700 cm^−1^ (Python 3.8, Anaconda 3)^41^. All spectra were normalized and maxima of the bands were identified with OriginPro 2019b from OriginLab.

### Equilibrium degree of swelling, gel yield and mesh size

To determine the equilibrium degree of swelling (*EDS*) and the gel yield (*Y*_g_), rectangular samples were punched out in the size of 1 cm × 2 cm after cross-linking. The *EDS* was calculated with the following equation:

1 $$EDS= \frac{{m}_{\mathrm{swollen}}-{m}_{\mathrm{dry}}}{{m}_{\mathrm{dry}}}$$

*m*_swollen_ is the mass of the hydrogels after swelling in water for 72 h, replacing the water every 24 h, and *m*_dry_ is the mass of the samples after drying under reduced pressure of approx. 50 mbar at 60 °C for 24 h (VDL 53, Binder GmbH).

The gel yield (*Y*_g_) was determined as follows:

2 $${Y}_{\mathrm{g}}= \frac{{m}_{\mathrm{dry}}}{{m}_{\mathrm{pol}}}$$

Here, *m*_pol_ is the mass of the samples directly after cross-linking.

For the estimation of the mesh size of PEGDA-0 samples, first the equilibrium polymer volume fraction *ϕ* of the hydrogels was calculated by:

3 $$\phi =\frac{{V}_{\mathrm{dry}}}{{V}_{\mathrm{swollen}}}=\frac{{m}_{\mathrm{dry}}/{\rho }_{\mathrm{PEGDA}}}{{(m}_{\mathrm{swollen}}-{m}_{\mathrm{dry}})/{\rho }_{\mathrm{H}20}+{m}_{\mathrm{dry}}/{\rho }_{\mathrm{PEGDA}}}$$

In this equation, *V*_dry_ is the volume of the dried hydrogel, *V*_swollen_ is the volume of the swollen hydrogel, *ρ*_PEGDA_ is the density of PEGDA (1.12 g cm^−3^)^42^ and *ρ*_H20_ is the density of water which was assumed to be 1.0 g cm^−3^. From *ϕ*, the molar mass *M*_c_ between cross-links of the hydrogel networks was estimated using the Flory-Rehner equation ^43,44^:

4 $$\frac{1}{{M}_{\mathrm{c}}}=\frac{2}{{M}_{\mathrm{n}}}-\frac{\frac{{\rho }_{\mathrm{H}20}}{{\rho }_{\mathrm{PEGDA}}}\cdot \left[\mathrm{ln}\left(1-\phi \right)+\phi +\chi \cdot {\phi }^{2}\right]}{{M}_{1}{\phi }_{0}\cdot \left[{\left(\frac{\phi }{{\phi }_{0}}\right)}^{1/3}- \frac{\phi }{{2\cdot \phi }_{0}}\right]}$$

Here, *M*_n_ is the number average molar mass of the PEGDA polymers before cross-linking, *χ* = 0.426 is the Flory–Huggins interaction parameter of PEGDA with water^45^, *M*_1_ = 18.01 g mol^−1^ is the molar mass of water, and *ϕ*_0_ is the polymer volume fraction in the hydrogels before swelling. For samples prepared in this study, no additional solvent was used during cross-linking and *ϕ*_0_ was 1. For estimation of the mesh size of hydrogels prepared by Tan et al*.*, their reported *ϕ*_0_ of 0.2 with an *EDS* of 9.27 was used^15^.

### Measurement of the isoelectric point

The pH-dependent curves of the zeta potential of the hydrogel surfaces were measured with an electrokinetic analyzer (Surpass™, Anton Paar GmbH). All samples were punched out in the size of 1 cm × 2 cm and were washed and swollen in 1 mM KCl for 72 h to equilibrate. After removing remaining solution with a filter paper, they were affixed on sample holders with double-sided tape (tesafix® 4965 original) and redundant hydrogel material was discarded. The sample holders were mounted in the adjustable gap cell, which was assembled in the electrokinetic analyzer. The freshly prepared electrolyte solution consisting of 1 mM KCl was purged with nitrogen gas as suggested by the manufacturer. The pH-dependent zeta potential was determined by measuring the streaming current in the pH-range of approximately 2.5 to 10.4 using 0.1 M HCl and 0.1 M KOH, measuring from acidic to basic pH, with a sample gap between 100 µm and 120 µm. The pH-value where the zeta potential was 0 mV was taken as the isoelectric point of the sample. Deionized water with a conductivity of 0.055 µS cm^−1^ (arium^®^ pro, Sartorius AG) was used.

### Water contact angle and free surface energy

The water contact angles (WCA) and free surface energy (SE) were measured with video analysis (DataPhysics Instruments GmbH, OCA 40). Samples were punched out in the size of 1 cm × 2 cm and WCA were measured with four drops of 2 µL deionized water with the sessile drop (SD) method. The videos were analyzed afterwards and the time point of 1 s was chosen for comparison. The same procedure was performed for hydrogels which were swollen in deionized water for 72 h.

The SE of the samples was determined by contact angle measurement with the three liquids deionized water (according to Ström et al.: SE = 72.8 mN m^−1^, polar portion (*σ*^*p*^) = 51.0 mN m^−1^, disperse portion (*σ*^*d*^) = 21.8 mN m^−1^)^46^, diiodomethane (according to Ström et al.: SE = 50.8 mN m^−1^, *σ*^*p*^ = 0.0 mN m^−1^, *σ*^*d*^ = 50.8 mN m^−1^)^46^ and ethylene glycol (according to Gebhardt: SE = 47.7 mN m^−1^, *σ*^*p*^ = 21.3 mN m^−1^, *σ*^*d*^ = 26.4 mN m^−1^)^47^. As before, a video was recorded and the values after 1 s were analyzed. The drop volume was also 2 µL and four drops were measured per sample. The SE was then determined with the Owens–Wendt-Rabel-Kälble (OWRK) model^48–50^.

For captive bubble (CB) measurements, hydrogels were swollen in deionized water for 72 h. Remaining water was removed with a filter paper, hydrogels were affixed on cover slips with double-sided tape (tesafix® 4965 original) and placed in a bowl filled with deionized water. The contact angle of an air bubble (10 µL) released by a J-shaped cannula was measured and the respective WCA was determined. Three bubbles were measured per sample.

### Antibacterial assay of the hydrogels

Potential antibacterial properties of the hydrogels were assessed according to ISO 22,196 with *Escherichia coli* (*E. coli*) DSM 1576. PEGDA-0 and PEGDA-2 samples, both n = 2, in the size of 2 cm × 2 cm, as well as plastic films in the appropriate size, were sterilized by UV treatment (Bio-Link BLX-312, Vilber) with 254 nm and 0.48 mW cm^−2^ for 20 min or 3 h, respectively. The bacteria were inoculated on LB agar overnight at 35 °C ± 1 °C and the pre-culture was grown overnight in LB medium at 35 °C ± 1 °C. The bacteria suspensions were diluted to a concentration of 6 × 10^5^ colony forming units (CFU) mL^−1^. 300 µL of the suspension were added on the sample surfaces and the plastic films were placed on top. The bacteria were washed off the surfaces and the films with 10 mL PBS either directly (0 h) or after 24 h incubation (24 h) at 35 °C ± 1 °C. The bacteria suspension was diluted in PBS in two concentrations and each was plated on LB agar (n = 3). The agar plates were incubated for 24 h at 35 °C ± 1 °C and the CFU were counted.

### Adsorption isotherms and kinetics of diclofenac in the hydrogels

Directly after preparation, samples with a diameter of 8 mm were punched out and weighed (*m*_pol_). The hydrogels were washed and swollen in deionized water for 24 h. The swollen hydrogels were immersed in 4 mL of aqueous diclofenac sodium salt (DSS) solution with initial concentrations *c*_0_ between 0 and 1 mg mL^−1^ for the determination of the adsorption isotherms and in 4 mL of DSS solution with a concentration *c*_0_ = 0.1 mg mL^−1^ for the adsorption kinetics. The hydrogels in DSS solution were kept under agitation at 45 rpm and 20 °C for 72 h for the adsorption isotherms and for a specific time for the adsorption kinetics. After this time, part of the supernatant was transferred into HPLC vials. The diclofenac concentrations *c*_e_ were determined by means of HPLC (Shimadzu Deutschland GmbH) using an isocratic flow of 1 mL min^−1^ of a solvent mixture consisting of 55% deionized water (with 0.05% TFA) and 45% acetonitrile. A volume of 10 µL was injected. All measurements were conducted at 40 °C. A reversed phase ReproSil™ XR C18 5 µm column (Dr. Maisch GmbH) was used for the separation. Calibration measurements were performed using twelve standard solutions of DSS with concentrations between 0 and 1 mg mL^−1^. Evaluation of the chromatograms was carried out by measuring the UV absorption at 275 nm. In order to determine the diclofenac concentration of each sample, the diclofenac peaks were integrated between a retention time of 9.1 min and 10.3 min. The amount *a*_*e*_ of diclofenac contained per mass of hydrogel was determined through the measured concentration *c*_*e*_ in the supernatant as

5 $${a}_{\mathrm{e}}=\frac{({c}_{0}-{c}_{\mathrm{e}})\cdot V}{{m}_{\mathrm{pol}}\cdot {M}_{\mathrm{d}}}$$

*M*_d_ is the molar mass of DSS (318.13 g mol^−1^). The resulting adsorption data were described with common adsorption models. With the Langmuir adsorption model, the concentration *q*_e_ of diclofenac adsorbed on the hydrogel polymer network is described by:

6 $${q}_{\mathrm{e}}={q}_{\mathrm{m}}\frac{{K}_{\mathrm{s}}\cdot {c}_{\mathrm{e}}}{1+{K}_{\mathrm{s}}\cdot {c}_{\mathrm{e}}}$$

*q*_m_ is the maximum adsorption capacity of the hydrogel polymer network, *K*_s_ is the ratio of the adsorption and desorption rate constants. Additionally, diclofenac is also present in the water that is the swelling medium of the hydrogel network, so the total concentration of diclofenac in the hydrogel is described by^51^:

7 $${a}_{\mathrm{e}}=\frac{{c}_{\mathrm{e}}\cdot {\varphi }_{\mathrm{H}2\mathrm{O}}}{{\rho }_{\mathrm{H}}}+{q}_{\mathrm{e}}$$

*φ*_Η2Ο_ is the water volume fraction (${\varphi }_{\mathrm{H}2\mathrm{O}}=1-\phi$) and *ρ*_H_ the density of the hydrogel. The partition coefficient *k* is defined as the ratio of adsorbate concentration in the hydrogel and of the equilibrium concentration in the supernatant:

8 $$k=\frac{{a}_{\mathrm{e}}\cdot {\rho }_{\mathrm{H}}}{{c}_{\mathrm{e}}}$$

Enhancement factors can be calculated as the ratio of the real measured partition coefficients and the ideal partition coefficients which equal the water volume fraction of the gels^52^.

9 $$E=\frac{k}{{\varphi }_{\mathrm{H}2\mathrm{O}}}$$

### Reduction of isothiouronium groups to thiols and their detection

Hydrogels were swollen in deionized water for at least 16 h. To reduce the isothiouronium groups to thiols, hydrogels were punched in the size of 1 cm × 2 cm and transferred into 5 mL 1 M sodium metabisulfite solution (Na_2_S_2_O_5_) and the solution was heated up to 60 °C for 5 h. These treated hydrogels are further called PEGDA-T hydrogels. Non-treated control samples were swollen in 5 mL fresh deionized water at RT for 5 h (PEGDA-NT hydrogels). After cooling down to RT, the supernatants were discarded and samples were shortly washed with 5 mL PBS. From this moment on all hydrogels were washed with PBS at least 5 × for 30 min until the pH-value of the washing solution was approx. 7.2.

Fluorescence staining with a maleimide dye was carried out after the hydrogels were kept in fresh PBS overnight. The hydrogels were punched out in discs with a diameter of 8 mm. After another washing step with PBS, the hydrogels were stained with the maleimide fluorophore Atto488 by immersing them in 2 mL of a 25 µM staining solution (obtained by diluting a 1 mM stock solution in DMSO 1:40 with deionized water) for 2 h at RT under the protection of light. Afterwards, hydrogels were shortly washed in PBS and then washed 7 × in PBS for 30 min and kept in PBS further on. All washing steps were conducted under the protection of light. A confocal laser scanning microscope (LSM) (LSM 710, AxioObserver, Carl Zeiss) was used to investigate the hydrogels with the objective Zeiss EC Plan-Neofluar 10x/0.30 M27. Microscope settings were kept identical for every sample and z-stacks were generated using the Argon 488 nm laser. The data was converted into a 2 D maximum intensity projection (MIP) and the mean fluorescence intensity of the MIP was determined with Fiji (2.1.0)^53^.

### Conjugation of horseradish peroxidase

After the washing steps with PBS, the PEGDA-0NT, PEGDA-0T, PEGDA-2NT and PEGDA-2T hydrogels were punched out in the size of 8 mm and placed in a 24 well plate. A maleimide-PEG_11_-biotin linker in a 250 mM stock solution in DMSO was further diluted to a concentration of 5 µM by addition of PBS. 500 µL of the solution were added to one half of the hydrogels, the other half was immersed in PBS. The hydrogels were agitated for 5 h at RT. Afterwards, they were shortly washed in PBS and then washed 7 × in PBS for 30 min and kept at 4 °C overnight. A solution of 2.5 µg mL^−1^ streptavidin-coupled horseradish peroxidase (SA-HRP) in PBS in a volume of 500 µL was added to each of the hydrogels for 5 h at 4 °C. The hydrogels were shortly washed in PBS and washed again 7 × for 30 min at 4 °C. 500 µL ABTS solution were added to each well and the absorption was measured at 405 nm for approx. 30 min. PEGDA-0NT hydrogels without the addition of the linker served as blank.

### Statistical analysis

One- or two-way analysis of variance (ANOVA) with Bonferroni post-hoc test were used for statistical analysis with OriginPro 2019b (OriginLab). For *p*-values < 0.05, mean values were considered to be significantly different. Each experiment, except for the antibacterial assay, was repeated independently for at least three times with independent sample preparations.

## Results and discussion

### Synthesis and characterization of isothiouronium-functional monomer AUITB

**Figure 1:**
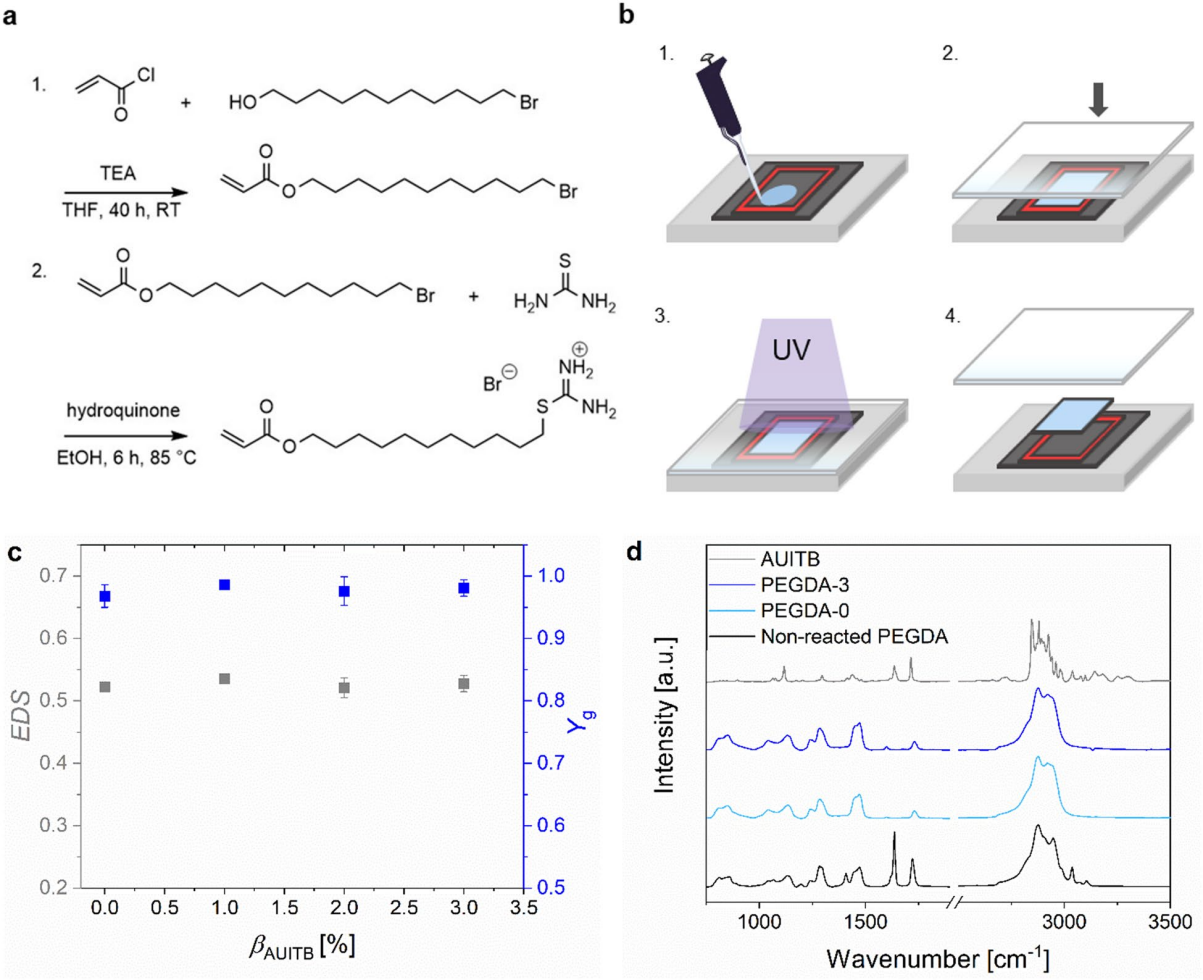
Hydrogel preparation and evaluation of the cross-linking process. (**a**) Synthesis of 2-(11-(acryloyloxy)undecyl)isothiouronium bromide (AUITB). First step: Synthesis of 11-bromoundecyl acrylate by acrylation of 11-bromo-1-undecanol with acryloyl chloride. Second step: Synthesis of AUITB by nucleophilic substitution with thiourea. TEA: triethylamine, THF: tetrahydrofuran, RT: room temperature, EtOH: ethanol. (**b**) Preparation of samples: 1. Pipetting the precursor solution on an activated silicon wafer. 2. Closing the mold with a quartz glass pane. 3. Cross-linking of the sample by UV-irradiation. 4. Removing the sample from the mold. (**c**) Equilibrium degree of swelling (*EDS*) in grey and gel yield (*Y*_g_) in blue of PEG-based hydrogels depending on the mass fraction *β*_AUITB_ of AUITB used during sample preparation. Hydrogels were swollen in deionized water for 72 h. Results are shown for PEGDA-0 (*EDS*: n = 10, *Y*_g_*:* n = 8), PEGDA-1 (*EDS*: n = 5, *Y*_g_*:* n = 3), PEGDA-2 (*EDS*: n = 6, *Y*_g_*:* n = 4) and PEGDA-3 (*EDS*: n = 5, *Y*_g_*:* n = 4) hydrogels. (**d**) Raman spectra of AUITB, unreacted PEGDA, a PEGDA-0 sample and a PEGDA-3 sample. Hydrogel samples were washed and dried before recording the spectra.

The synthesis of the monomer AUITB is based on a two-step reaction (Fig. 1a). In the first step, 11-bromoundecyl acrylate was synthesized by the acrylation of 11-bromo-1-undecanol with acryloyl chloride. As a by-product, 11-hydroxyundecyl acrylate was formed. However, this by-product, lacking the electrophilic bromoalkane moiety, was unreactive in the reaction conditions used in the second step of the reaction, therefore no further purification was performed. In the second step, AUITB was synthesized by a nucleophilic substitution with thiourea. The spectral data indicated a successful synthesis of the monomer AUITB. The ^1^H NMR signals of the isothiouronium group at 8.99 ppm were in accordance with previous reports^54–57^. The Raman spectrum showed the typical stretching vibration of the C-H bond in the alkyl chain between 2800 and 3000 cm^−1^ (Fig. 1d). The band at 1714 cm^−1^ was associated with the *α,β*-unsaturated ester in the molecule. The band at 1637 cm^−1^ was related to the stretching vibration of the C = C double bond. In the same wavenumber range also the band of the N–H bond is localized^58^. AUITB was soluble in chloroform, dimethyl sulfoxide, ethanol and also in neat PEGDA, however was insoluble in water. Solutions of AUITB in PEGDA were therefore used for subsequent hydrogel preparation.

### Preparation and characterization of isothiouronium-functional hydrogels

PEGDA samples with and without the monomer AUITB were prepared by radical cross-linking in molds (Fig. 1b). First, it was evaluated if the integration of AUITB interfered with the cross-linking process of PEGDA. For this purpose, the equilibrium degrees of swelling (*EDS*) and the gel yields (*Y*_g_) were examined. Figure 1c shows the *Y*_g_ and *EDS* as a function of the mass fraction *β*_AUITB_ of AUITB used during sample preparation for the samples PEGDA-0, PEGDA-1, PEGDA-2 and PEGDA-3. The *Y*_g_ as well as the *EDS* were comparable for the different hydrogel compositions. The mean values of *Y*_g_ ranged between 0.97 and 0.99, indicating an almost quantitative integration of all starting materials into the polymer network for all samples. The *EDS* was between 0.52 and 0.54, very similar to the *EDS* of 0.49 reported by Mellott et al. for spheres prepared with PEGDA (*M*_n_ = 575 g mol^−1^)^14^.

It is interesting to note that the introduction of the charged monomer AUITB did not alter the swelling properties significantly, in contrast to our expectations and to previous studies with monomers carrying ionic groups. For example, Tan et al. prepared PEGDA (*M*_n_ = 4000 g mol^−1^) hydrogels with the monomer 2-(methacryloyloxy)ethyltrimethylammonium chloride (MAETAC) having a quaternary ammonium group^15^. Their solutions before curing contained 20% w/v PEGDA leading to an *EDS* of 9.3 without MAETAC and to an *EDS* of 15.6 with approx. 20.8 wt% MAETAC relative to the mass of PEGDA. However, there are important differences between our experiments and the data described by Tan et al*.* With up to 3 wt% AUITB in our precursor solution, the fraction of the functional monomer is much lower. The molar ratio of AUITB and PEGDA was therefore up to 5.7%. Taking into account the two polymerizable groups per PEGDA molecule, this results in a theoretical fraction of isothiouronium-functionalized repeating units in the polyacrylate backbone of 2.8%. Also, in contrast to MAETAC, AUITB has a hydrophobic alkyl chain which will probably rather decrease the *EDS*. Additionally, we consider one of the key differences the cross-link density of the pure PEGDA hydrogels. Using Eq. 4 to estimate the molar mass between cross-links, one obtains a value of 48 g mol^−1^ for our samples, which is close to one repeating unit of the PEG backbone, in contrast to a value of 1145 g mol^−1^ for the samples of Tan et al. Thus, our hydrogels contain densely cross-linked polymer networks which offer only very limited possibilities for the network to expand, also after addition of AUITB. Therefore, the *EDS* data in combination with the *Y*_g_ data suggest that AUITB did not interfere with the cross-linking process of PEGDA for the tested concentrations.

In order to confirm this finding, Raman spectra were recorded of AUITB, unreacted PEGDA, a PEGDA-0 sample and a PEGDA-3 sample. The spectra are depicted in Fig. 1d.

All spectra had the characteristic bands of the stretching vibrations of the C-H bond between 2600 and 3000 cm^−1^. The band > 3000 cm^−1^ in the spectrum of the non-reacted PEGDA results from the C-H stretching vibration at the double bond. This spectrum also had a strong band with a peak at 1637 cm^−1^, which can be assigned to the C = C double bond. The spectra of the cross-linked PEGDA-0 and PEGDA-3 samples were comparable and showed a nearly disappeared band around 1600 cm^−1^. This indicates that the cross-linking of the samples was successful, including the consumption of the C = C double bond, and that the integration of the monomer in the precursor solution did not interfere with the cross-linking process. Unfortunately, in the PEGDA-3 spectrum, no direct evidence was visible that proves the successful integration of AUITB, probably due to the rather small *β*_AUITB_. Summarizing, the Raman spectra also point out that cross-linking of PEGDA hydrogels was not hampered by AUITB addition.

### Functionalities of isothiouronium-containing hydrogels

**Figure 2:**
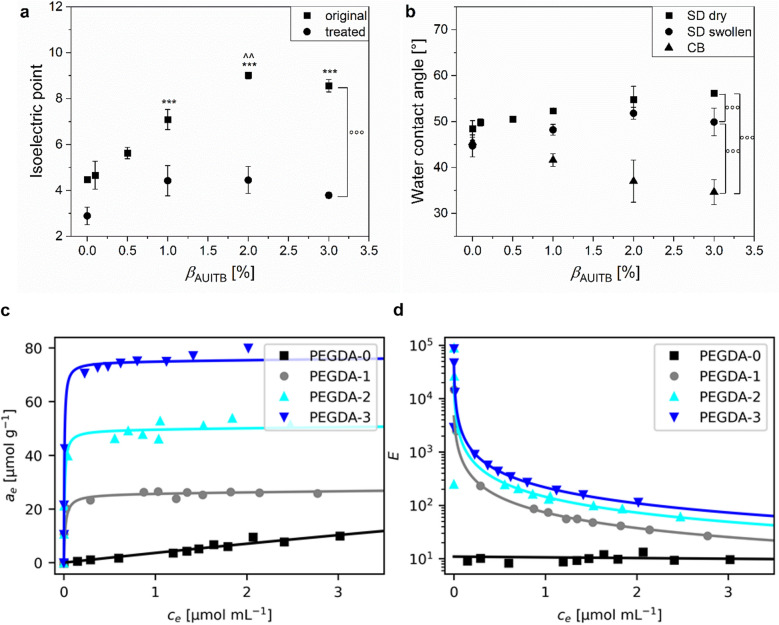
Characterization of the isothiouronium-functional hydrogels. (**a**) Isoelectric points (IEP) depending on the mass fraction *β*_AUITB_ of AUITB used during sample preparation. Results are shown for PEGDA-0, PEGDA-0.1, PEGDA-0.5, PEGDA-1, PEGDA-2 and PEGDA-3 hydrogels (n = 3). Hydrogels were either swollen in 1 mM KCl after cross-linking (original) or treated with 1 M Na_2_S_2_O_5_ and kept in 1 mM KCl afterwards (treated). Explanation of symbols: *** significantly different to PEGDA-0 hydrogels (p < 0.001); ^^ significantly different to PEGDA-1 hydrogels (p < 0.01), °°° significant differences between sample treatments (p < 0.001). (**b**) Water contact angles depending on *β*_AUITB_ of dry and swollen samples, measured by the sessile drop (SD) or captive bubble (CB) method (n = 3, SD method PEGDA-2 n = 4). Explanation of symbols: °°° significant differences between methods (p < 0.001). (**c**) and (**d**) Diclofenac adsorption in isothiouronium-functional hydrogels. (**c**) Diclofenac concentrations *a*_e_ adsorbed within hydrogels PEGDA-0, PEGDA-1, PEGDA-2 and PEGDA-3, determined for different equilibrium supernatant concentrations *c*_*e*_ of diclofenac. The experimental data points were fitted with a modified Langmuir model according to Eq. 7 (solid lines). (**d**) Enhancement factors *E* of diclofenac within the tested samples. The data points were calculated using Eq. 9 with the experimental values of *a*_e_ and *c*_e_, and the solid lines were calculated with the same equation by expressing *a*_e_ with the fit results shown in Table 1.

In order to further evaluate if the functionalization of the PEGDA hydrogels with AUITB was detectable and successful, their isoelectric points (IEP), *i.e.* the pH-value where the net charge is zero, were examined by measuring the zeta potential as a function of pH value (Figure SI 4). In Fig. 2a (‘original’), the IEPs of samples with *β*_AUITB_ between 0 wt% and 3 wt% are shown. The hydrogels were swollen in 1 mM KCl after cross-linking for three days to facilitate the measurements, but apart from that received no chemical treatment. Generally, the IEP of the hydrogels increased with increasing *β*_AUITB_. Their IEPs ranged between 4.5 ± 0.1 (PEGDA-0) and 9.0 ± 0.1 (PEGDA-2). For the non-functionalized surface of the PEGDA-0 samples, an IEP of approx. 4 was expected^59^. It has been reported before that PEG-based hydrogels have their IEP in this pH-range^60–62^ probably caused by the adsorption of hydroxide ions on the surface^61,63,64^. With increasing *β*_AUITB_, the IEP shifted to values in the basic pH-range. If a solid surface only has one functional group, the IEP of this surface is reported to be strongly related to the p*K*_a_-value of this group^59^. The p*K*_a_-value of isothiouronium groups is expected to be in the basic region^65–67^. For isothiouronium-functionalized nanoparticles, an IEP of 10.2 has been reported^27^. These findings are in accordance with the IEP measured for the higher *β*_AUITB_ of 2 wt% and 3 wt%. Hence the results suggest that the functionalization of the PEGDA hydrogels led to a surface charge and that the amount of isothiouronium groups within the samples correlated with *β*_AUITB_ used during preparation.

**Table 1:** Adsorption capacity *q*_m_ and equilibrium constant *K*_s_ describing the adsorption of diclofenac on PEGDA hydrogels with and without isothiouronium functionalization. The values were obtained by nonlinear regression of the adsorption isotherms (Fig. 2c and d) with Eq. 7.

Sample	*q*_m_ [µmol g^−1^]	*K*_s_ [mL µmol^−1^]
PEGDA-0	102 ± 246	0.03 ± 0.09
PEGDA-1	25.7 ± 1.3	63 ± 23
PEGDA-2	49.6 ± 1.9	131 ± 49
PEGDA-3	74.9 ± 1.4	192 + 35

In order to evaluate the effect of AUITB addition to the hydrogels further, contact angle measurements were performed. It is conceivable that due to the amphiphilic structure of AUITB, either more hydrophilic or more hydrophobic surface properties are generated upon AUITB integration. Therefore, first the water contact angles (WCA) of dry and swollen samples in contact with air were measured with the sessile drop (SD) method (Fig. 2b). For dry PEGDA-0 samples, a WCA of 48.4° ± 1.8° was measured whereas the PEGDA-3 samples had a WCA of 56.2° ± 0.4°. Additionally, swollen PEGDA-0 hydrogels had a WCA of 44.7° ± 2.4° and swollen PEGDA-3 hydrogels a WCA of 49.9° ± 3.0°. Thus, there was a tendency towards increasing WCAs with increasing *β*_AUITB_, implying more hydrophobic surfaces, probably caused by the hydrophobic alkyl chain of AUITB. This result was supported by measuring the surface energies (SE) of the dry samples (Figure SI 5). All samples had a comparable SE between 51 and 53 mN m^−1^, enabling the comparison of the polar and disperse portions. A tendency towards decreasing polar portions was detected with increasing *β*_AUITB_. PEGDA-3 samples had polar portions of 12.7 mN m^−1^ ± 0.2 mN m^−1^ compared to PEGDA-0 samples with 15.6 mN m^−1^ ± 0.6 mN m^−1^.

However, interestingly a different result was obtained when measuring the WCAs with the captive bubble (CB) method during which the samples are submerged in water. The CB measurements resulted in similar contact angles of the PEGDA-0 hydrogels compared to the SD measurements of swollen PEGDA-0 hydrogels with 45.4° ± 0.5°. In contrast to the SD data, the WCA decreased with increasing *β*_AUITB_, so that PEGDA-3 hydrogels had a WCA of 34.6° ± 2.7°. We ascribe this observation to a changed orientation of the isothiouronium groups in samples submerged in water. This is possible due to the isothiouronium groups being linked to the flexible alkyl chain which can change conformation depending on the surrounding medium. In contact with the non-polar air, the alkyl chain is exposed to the sample surface, whereas in contact with water the ionic isothiouronium group is presented. A similar effect of the reorientation of side chains induced by the surrounding medium was reported before by Yokoyama et al.^68^. They mixed a block copolymer of deuterated polystyrene (dPS) and 2-[2-(2-methoxyethoxy)-ethoxy]ethyl methacrylate (PME3MA) with polystyrene (PS) and measured decreased advancing and receding contact angles and higher hysteresis with increasing concentration of dPS-PME3MA in PS. They hypothesized this effect is due to the surface reconstruction of the hydrophilic parts of PME3MA, which can form a brush layer on PS. When exposed to air the methyl termini were located at the surface^68^. These reorganizations of the surface probably take place to lower the interfacial free energy^68–70^.

In fact, our method used to prepare the samples involved contact of the analyzed surface to a hydrophilic silicon surface. The reasoning behind this was to allow the isothiouronium groups to be present on the sample surfaces. The WCA measurements now confirm this approach. A similar method was previously described by Bongiovanni et al. who prepared acrylic films and added acrylic monomers with different lengths of alkyl chains to an epoxyacrylic resin^71^. While cross-linking those films, one side was exposed to an inert atmosphere and the other one to a glass substrate. They reported an asymmetric distribution of the monomers, depending on the alkyl chain length and monomer concentration, and found increasing advancing contact angles on the film side orientated towards the inert atmosphere. Thus, we conclude that the combination of our sample preparation procedure and a polar surrounding medium like water makes the isothiouronium groups accessible on the sample surfaces, as also suggested by the IEP data described above. Moreover, the results also indicate a comparable surface density of isothiouronium groups for PEGDA-2 and PEGDA-3 hydrogels.

Additionally, potential antibacterial properties of the hydrogel surfaces were assessed for PEGDA-2 and PEGDA-0 hydrogels with the bacterial reference strain *E. coli* DSM 1576 according to ISO 22,196 under static conditions (Figure SI 6). Comparable CFU mL^−1^ of *E. coli* were seeded on the hydrogel surfaces with (185 ± 41) CFU mL^−1^ for PEGDA-0 and (177 ± 22) CFU mL^−1^ for PEGDA-2 hydrogels. After 24 h of contact time with the samples (520 ± 117) CFU mL^−1^ were counted on PEGDA-0 hydrogels and (395 ± 49) CFU mL^−1^ on PEGDA-2 hydrogels, showing a slight tendency of less bacteria on PEGDA-2 hydrogels. The effect of isothiouronium groups on bacteria was investigated before by Cohen et al*.* using a procedure under dynamic conditions^27^. They added polyisothiouronium methylstyrene nanoparticles to bacteria suspensions, including *E. coli*, and demonstrated antibacterial properties of the nanoparticles. Our data do not suggest a distinct antibacterial effect. Since the bacteria needed to be washed off the sample and film surfaces before they were counted, bigger errors were expected and also determined. Additionally the number of available isothiouronium groups on the hydrogel surfaces is unknown and probably lower compared to the functionalized nanoparticles.

As mentioned above, isothiouronium-functionalized materials may be used as adsorbers for heavy metal ions. In this contribution, we wanted to evaluate the potential of the isothiouronium-functionalized hydrogels for the adsorption of pharmaceuticals like diclofenac, which was offered in its ionic form of DSS. For this purpose, adsorption isotherms, *i.e.* the concentration *a*_e_ of diclofenac inside the hydrogels as a function of the equilibrium concentration *c*_e_ in the supernatant (Eq. 7), were measured for PEGDA-0, PEGDA-1, PEGDA-2 and PEGDA-3, as shown in Fig. 2c. The adsorption time to reach equilibrium conditions was found to be 72 h, as shown in Figure SI 7. The overall shapes of the isotherms of isothiouronium-functionalized samples were similar. The results show steep increases of *a*_e_ with increasing *c*_e_ for all AUITB-containing hydrogels at low *c*_e_. At higher *c*_e_ values, the isotherms flattened out and approached a plateau. It was also evident that the maximum *a*_e_ values increased with AUITB concentration. For a quantitative understanding, the isotherms were fitted using Eq. 7 and the adsorption capacities *q*_m_, *i.e.* the level of the plateau, and the equilibrium constants *K*_s_ were obtained (Table 1). The values for *q*_m_ ranged between 25.7 and 74.9 µmol g^−1^, increasing proportionally with *β*_AUITB_. This observation is consistent with the assumption that indeed the isothiouronium groups cause the adsorption of diclofenac within the samples, probably by means of an ion exchange. This conclusion is further supported by the adsorption isotherm of PEGDA-0 which is almost linear without showing any plateau. This is indicative of unspecific adsorption due to the absence of isothiouronium groups. Also, the maximum *a*_e_ value was 9.9 µmol g^−1^ for the unfunctionalized samples, thus being much lower than for all AUITB containing samples. Note that the fit results for PEGDA-0 in Table 1 were included for the sake of completeness, but are not meaningful due to the large standard errors.

A similar picture is found by looking at the enhancement factors *E* of diclofenac in the hydrogels, *i.e.* the ratio of diclofenac concentrations inside the hydrogel and the supernatant (Fig. 2d). For PEGDA-1, PEGDA-2 and PEGDA-3 hydrogels, maximum enhancement factors of 14,911, 89,923 and 85,461 were achieved, whereas for the non-functionalized PEGDA-0 hydrogels a maximum value of 239 was determined. In fact, the large enhancement factors give rise to the conclusion that the isothiouronium functionalization results in a strong interaction with the diclofenac anion while the adsorption capacity of the hydrogels can be tuned by varying the amount of AUITB monomer. The strong affinity of the isothiouronium groups to diclofenac is also evident when comparing the *q*_m_ and *K*_s_ values from Table 1 with results reported before for diclofenac adsorption. Concerning *q*_m_, a higher value of 344.8 mg g^−1^ (1083 µmol g^−1^) was found for example by Liu et al. with polymeric nanoparticles prepared with the positively charged monomer MAETAC^25^, or of 129.42 mg g^−1^ (406 µmol g^−1^) by Mahmoodi et al*.* with melamine-functionalized graphene oxide-chitosan hydrogels^72^. This can simply be explained by the different concentration of adsorption sites, as also evident from the proportional increase of *q*_m_ with *β*_AUITB_. A higher affinity is connected to a higher *K*_s_ value. Indeed, the value for PEGDA-3 samples of 192 mL µmol^−1^ surpasses the values of 0.1663 L mg^−1^ (52.9 mL µmol^−1^) and 0.336 ppm^−1^ (107.0 mL µmol^−1^) reported before by Liu et al.^25^ and Mahmoody et al.^72^ The isothiouronium groups present in the hydrogels in our study therefore show a higher affinity to diclofenac than the functional groups in the previous reports.

### Functionalities of thiol-containing hydrogels

Isothiouronium groups are synthetic precursors of thiols, as explained in the introduction. Therefore, we treated the isothiouronium-functionalized samples with sodium metabisulfite to reduce them to thiols. The thus treated samples are denoted with a “T” in their sample names. Control samples which underwent the same washing steps, but omitting sodium metabisulfite in the solutions, are denoted with a “NT” in their sample names. The effect of the treatment was first assessed by measuring the IEP of the samples (Fig. 2a). Indeed, the IEPs decreased drastically for the treated and functionalized samples to a similar level like the PEGDA-0 samples. This demonstrates that the isothiouronium groups were converted to neutral moieties during the reaction, consistent with the presence of thiols.

**Figure 3:**
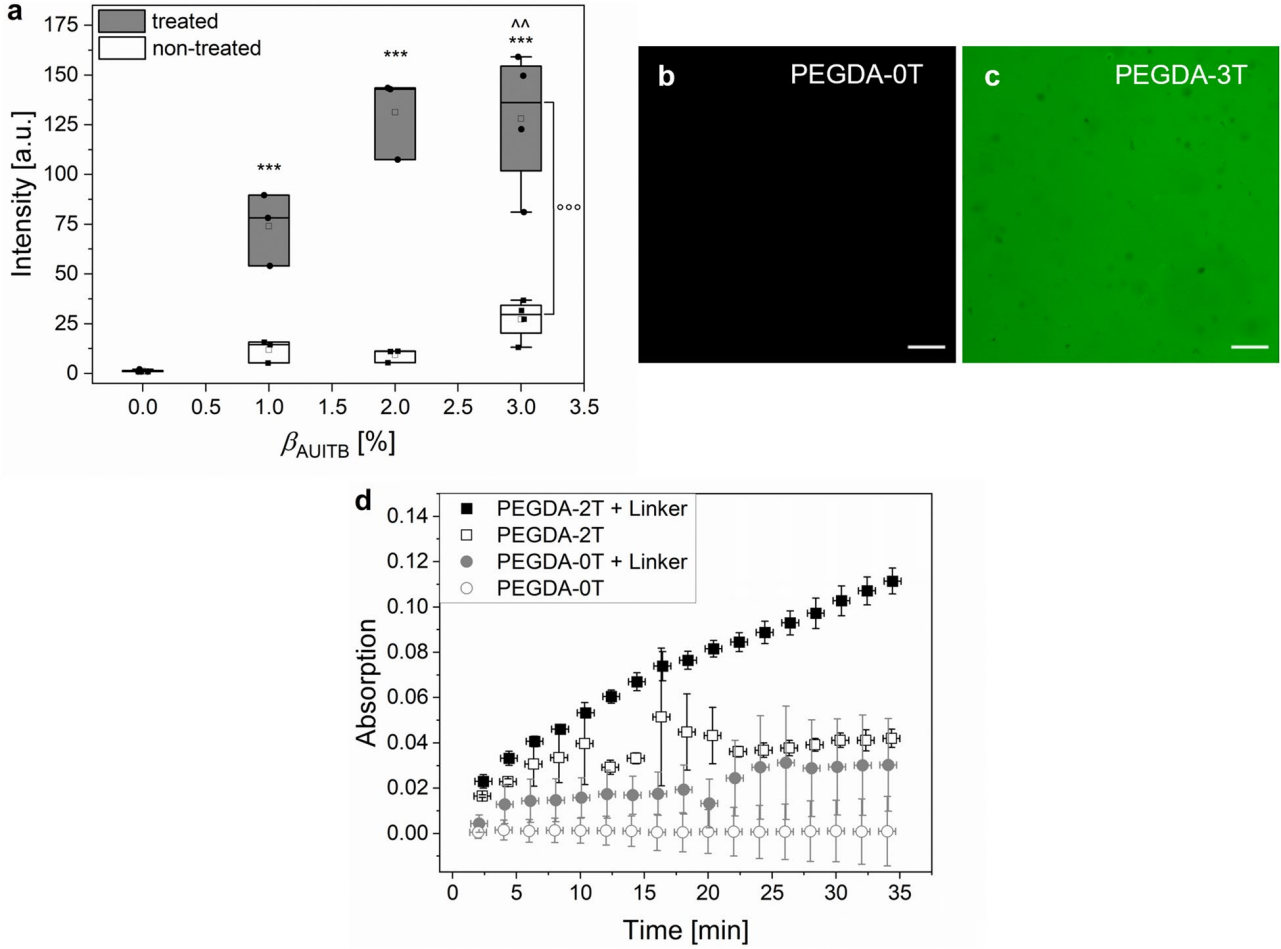
Functionalities of thiol-containing PEG-based hydrogels. (**a**) Fluorescence intensity measured for hydrogels with isothiouronium (non-treated) and thiol functionalization (treated) after staining with Atto488 maleimide as a function of the mass fraction *β*_AUITB_ of AUITB used during hydrogel preparation. Treated samples were chemically reduced with sodium metabisulfite (Na_2_S_2_O_5_) and non-treated were kept in water. The boxes show the 25th and 75th percentiles. The horizontal line in the boxes is the median and the square is the mean. (PEGDA-0, PEGDA-3: n = 4, PEGDA-1, PEGDA-2: n = 3). Explanation of symbols: *** significantly different to PEGDA-0 hydrogels (p < 0.001); ^^ significantly different to PEGDA-1 hydrogels (p < 0.01), °°° significant differences between sample treatments (p < 0.001). (**b**, **c**) Exemplary fluorescence images of stained hydrogels. (**b**) PEGDA-0T hydrogel. (**c**) PEGDA-3T hydrogel. Scale bar 100 µm. (**d**) Time dependent absorption measurements of the conversion of ABTS substrate to the green ABTS^+^ by horseradish peroxidase linked to the hydrogels. PEGDA-0NT hydrogels without the addition of the linker served as blank. Significant effects were found for the means in sample composition and time, the interaction of sample composition and time was significant as well.

In a second step, the reactivity of the treated samples was assessed by fluorescence staining of the hydrogels. Thiols were stained via a thiol-Michael reaction with the fluorescence dye Atto488 maleimide. In Fig. 3a, the fluorescence intensity of the treated and control samples is depicted. Figure 3b and c show representative fluorescence images after staining of the PEGDA-0T and PEGDA-3T hydrogels. Representative images after staining of all other tested hydrogel compositions are listed in Figure SI 8.

Generally, the fluorescence intensity after staining of treated samples increased with the mass fraction *β*_AUITB_ of AUITB used for sample preparation (Fig. 3a), whereas the non-treated samples still containing the isothiouronium groups were stained to a much smaller extent. This was also visually observed by a color change of the hydrogels (Figure SI 9). Also, both the treated and non-treated PEGDA-0 samples showed no staining. We explain this with the reactivity of the formed thiols in the treated, formerly isothiouronium-functionalized samples towards the fluorescence dye, resulting in covalent attachment, which is possible neither for the non-treated nor the treated PEGDA-0 samples. The small fluorescence intensity of the non-treated samples is ascribed to electrostatic interaction of the negatively charged dye and the isothiouronium groups. Taken together, the observations are a strong indication that indeed thiols were formed that were fully accessible and reactive, thus making them suitable to be used in further bioconjugation reactions. Also, the amount of available thiols for coupling increases with *β*_AUITB_. Indeed, it was reported before that isothiouronium groups can be reduced successfully to thiols with sodium metabisulfite^32^.

In a next step, the thiol-functionalized hydrogels were used to conjugate the enzyme horseradish peroxidase (HRP). This was achieved in a two-step process by first coupling a biotin-functional linker via a thiol-Michael reaction to the samples, and subsequently using the biotin-streptavidin interaction to bind a streptavidin-HRP conjugate (SA-HRP), a strategy similar to a report by Koch et al.^38^. In order to distinguish between unspecific adsorption of SA-HRP on the hydrogels and the desired bioconjugation reaction, control experiments without the linker were performed. The success of the reaction was assessed by conversion of ABTS to the green ABTS^+^ radical cation which was measured photometrically (Fig. 3d). The PEGDA-2T hydrogels, which were first agitated in linker solution and afterwards in enzyme solution (PEGDA-2T + Linker), had the highest absorption of 0.111 after 34 min in ABTS substrate solution. The PEGDA-2T hydrogels without the linker had a lower absorption of 0.042 at the same time point. The PEGDA-0T hydrogels had an absorption of 0.030 with and 0.001 without the linker at 34 min. As expected, the PEGDA-2T + Linker hydrogels had the highest absorption. Due to their thiols, the maleimide linker was covalently bound to the hydrogels, which was used in the next step to bind the streptavidin-coupled enzyme with biotin. Without the linker, the PEGDA-2T hydrogels could only unspecifically bind the SA-HRP and therefore the absorption was lower. The PEGDA-0T hydrogels had the lowest absorption, demonstrating the low unspecific protein adsorption on PEGDA hydrogels. The PEGDA-2NT hydrogels, containing isothiouronium groups, reached absorption values of 0.081 with and 0.070 without the linker, showing again an interaction between the isothiouronium groups and the maleimides and also an effect of the groups regarding protein adsorption (data shown in Figure SI 10). Summarizing, it was demonstrated that the thiol-functionalized hydrogels can serve as an anchor for further functionalization and in this case for bioconjugation reactions.

## Conclusions

We successfully demonstrated that isothiouronium groups can be introduced into hydrogels by simple radical copolymerization of the isothiouronium-functional monomer AUITB with PEGDA. The isothiouronium groups are fully accessible on the sample surface, especially in polar media, while they orientate themselves towards the sample bulk in contact with air. Furthermore, the isothiouronium groups are also fully accessible within the sample bulk, making the samples highly effective in the adsorption of diclofenac. Generally, the sample properties changed monotonically with the mass fraction of AUITB used during sample preparation, allowing a precise control of material properties. Finally, the isothiouronium groups can be easily reduced to thiols, thus harnessing the full potential of the versatile thiol reactivity for bioconjugation reactions, such as enzyme immobilization. In conclusion, the results show that isothiouronium functionalization offers an attractive and simple way to introduce chemical functionality into hydrogels.

## Supplementary Information

Supplementary Information.

## Data Availability

All data is available from the corresponding author upon reasonable request.
