# Supplementary material for: Preparation of multifunctional hydrogels with accessible isothiouronium groups via radical cross-linking copolymerization
Source: Sci Rep. 2023 Jun 26;13:10361. doi: 10.1038/s41598-023-36956-x (PMC10293292; doi:10.1038/s41598-023-36956-x)
Supplement: Supplementary file 1 — Supplementary Information. [file 41598_2023_36956_MOESM1_ESM.docx]

Supporting Information for

**Preparation of multifunctional hydrogels with accessible isothiouronium groups via radical cross-linking copolymerization**

Jana Grübel^1^, Vanessa L. Albernaz^1^, Anastasia Tsianaka^1^, Corinna O. Jauch^1^, Silia Quirin^1^, Christian Kerger^2^, Christina G. Kohl^2^, Anke Burger-Kentischer^2^, Günter E. M. Tovar^1,2^, Alexander Southan^1,^^[[1]](#footnote-1)^

^1^Institute of Interfacial Process Engineering and Plasma Technology IGVP, University of Stuttgart, Nobelstr. 12, 70569 Stuttgart, Germany. E-Mail: southan@is.mpg.de.

^2^Fraunhofer Institute for Interfacial Engineering and Biotechnology IGB, Nobelstr. 12, 70569 Stuttgart, Germany. E-Mail: guenter.tovar@igb.fraunhofer.de.

Table of Contents

[1. NMR spectra of synthesized products 2](#_Toc134617018)

[2. Hydrogel preparation 4](#_Toc134617019)

[3. Zeta potential measurements of isothiouronium-functional hydrogels 5](#_Toc134617020)

[4. Surface energy of isothiouronium-functional hydrogels 6](#_Toc134617021)

[5. Evaluation of an antibacterial effect of isothiouronium-functional hydrogels 6](#_Toc134617022)

[6. Adsorption kinetics of diclofenac in isothiouronium-functional hydrogels 7](#_Toc134617023)

[7. Fluorescence staining of thiols 7](#_Toc134617024)

[8. Substrate conversion on isothiouronium-functional hydrogels 8](#_Toc134617025)

# NMR spectra of synthesized products


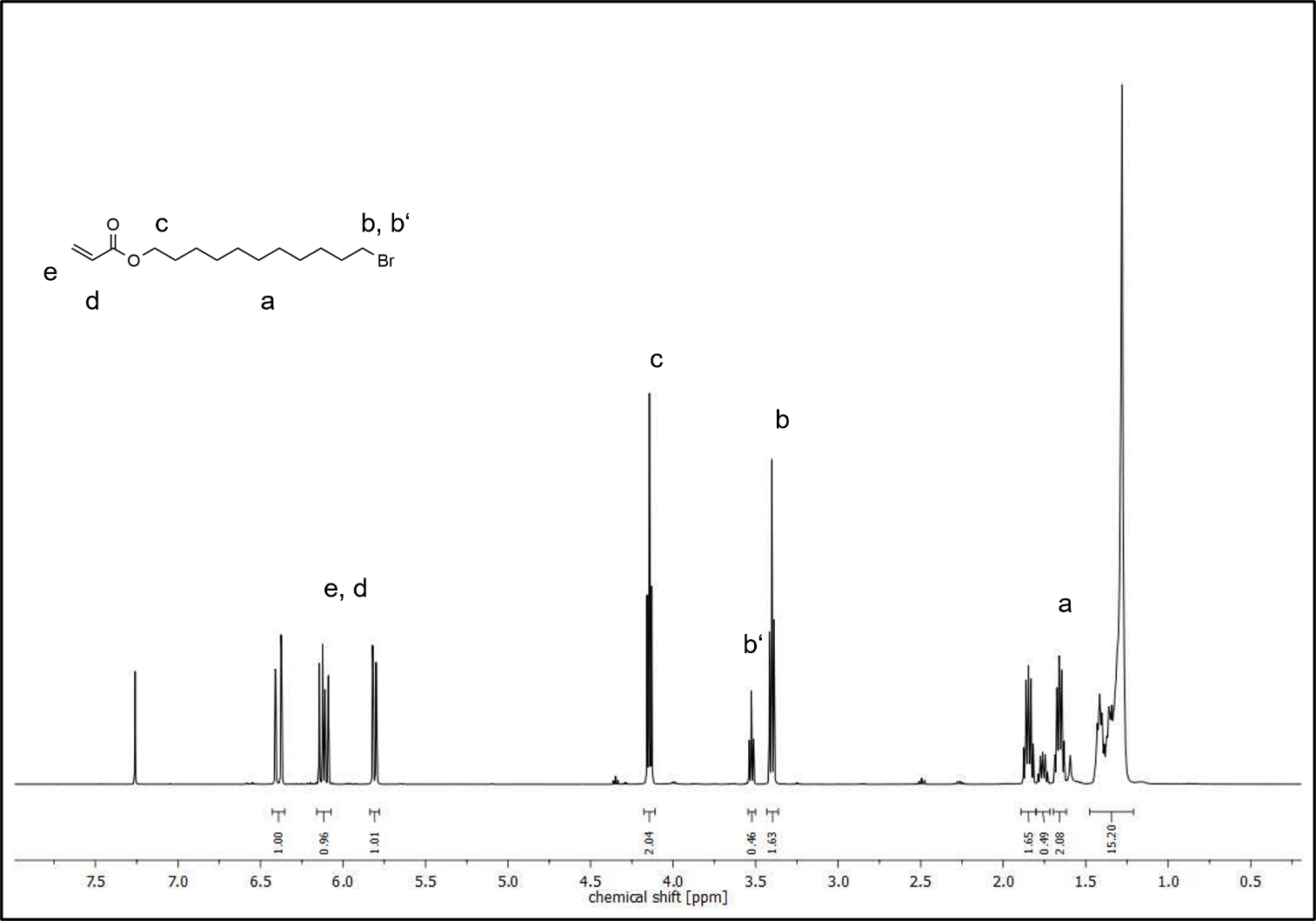


Figure SI 1. ^1^H NMR spectrum of 11-bromoundecyl acrylate.


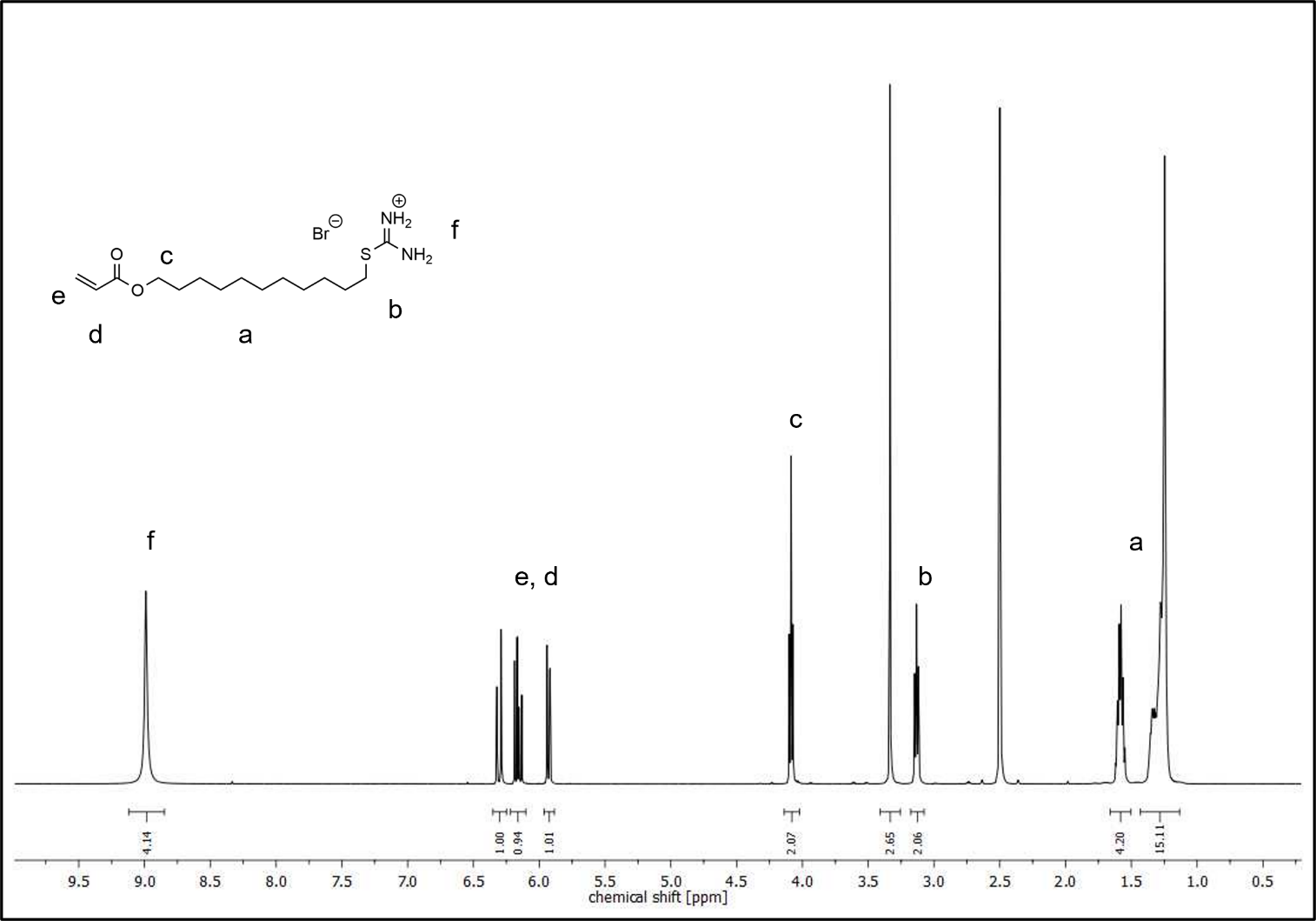


Figure SI 2. ^1^H NMR spectrum of AUITB.


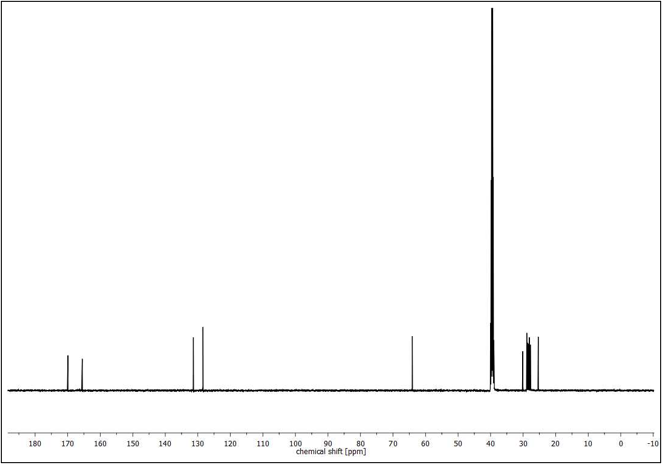


Figure SI 3. ^13^C NMR spectrum of AUITB.

# Hydrogel preparation

Table SI 1. Composition of the hydrogel precursor solution for sample preparation, given in mass fractions of PEGDA (*β*_PEGDA_), AUITB (*β*_AUITB_) and the photoinitiator Irgacure 2959 (*β*_PI_).

| ***β*_PEGDA_ [wt%]** | ***β*_AUITB_ [wt%]** | ***β*_PI_ [wt%]** |
| --- | --- | --- |
| 99.5 | 0.0 | 0.5 |
| 99.4 | 0.1 | 0.5 |
| 99.0 | 0.5 | 0.5 |
| 98.5 | 1.0 | 0.5 |
| 97.5 | 2.0 | 0.5 |
| 96.5 | 3.0 | 0.5 |

# Zeta potential measurements of isothiouronium-functional hydrogels


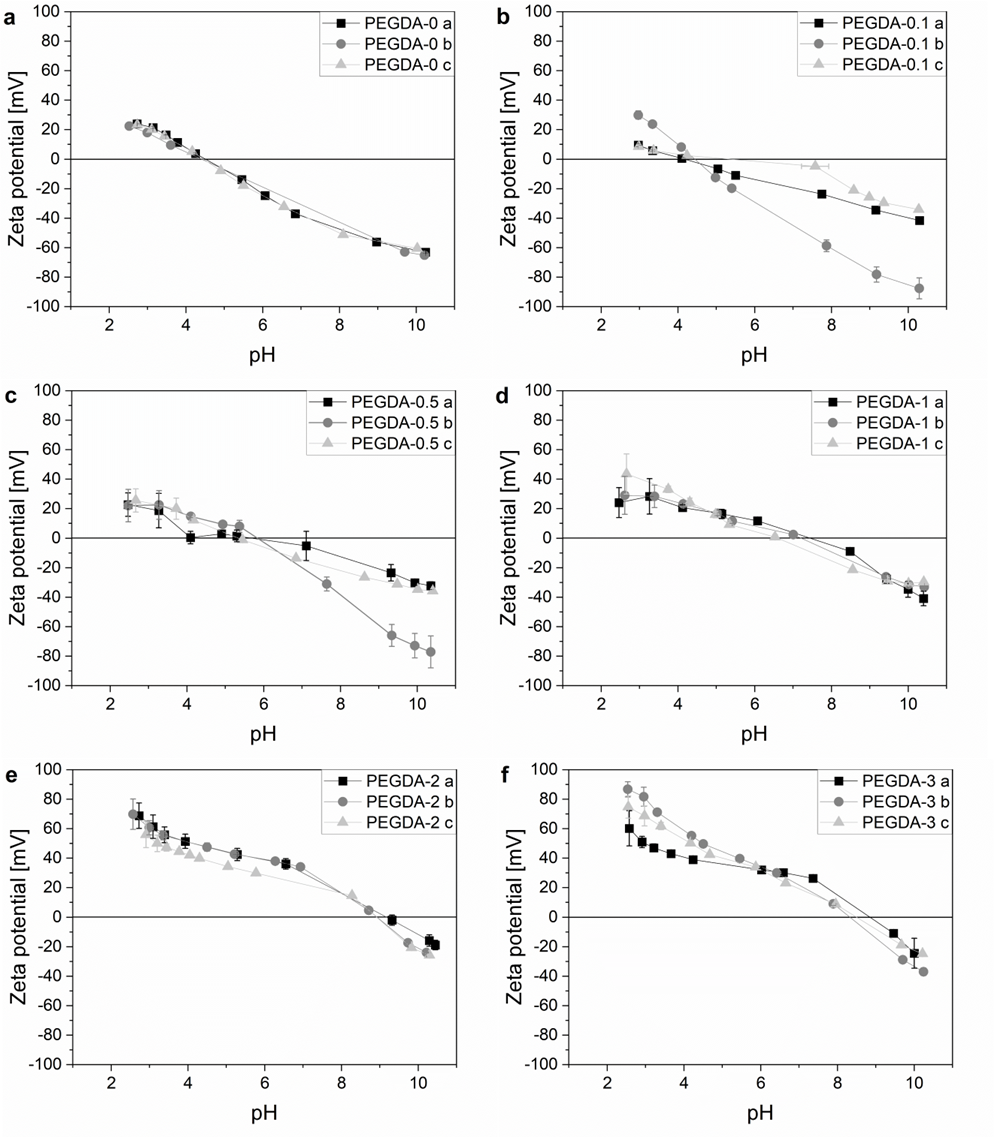


Figure SI 4. Zeta potential measurements of isothiouronium-functional hydrogels. The zeta potential as a function of the pH value is shown. a) PEGDA-0 hydrogels. b) PEGDA-0.1 hydrogels. c) PEGDA-0.5 hydrogels. d) PEGDA-1 hydrogels. e) PEGDA-2 hydrogels. f) PEGDA-3 hydrogels.

# Surface energy of isothiouronium-functional hydrogels





Figure SI 5. Surface energy of dry samples with its polar and disperse portions, measured with the sessile drop method (n = 3, PEGDA-2 n = 4). Explanation of symbols: **/* significantly different to PEGDA-0 (p < 0.01/0.05), ° significantly different to PEGDA-0.1 (p < 0.05), ^ significantly different to PEGDA-1 (p < 0.05).

# Evaluation of an antibacterial effect of isothiouronium-functional hydrogels





Figure SI 6. Colony forming units (CFU) mL^-1^ of *E. coli* on PEGDA-0 and PEGDA-2 hydrogels after 0 h and 24 h sample contact (n = 2).

# Adsorption kinetics of diclofenac in isothiouronium-functional hydrogels





Figure SI 7. Kinetics of diclofenac adsorption within isothiouronium-functional hydrogels. The diclofenac concentration *c*_e_ in the supernatant was measured at specific time points. After 72 h, equilibrium conditions were reached.

# Fluorescence staining of thiols


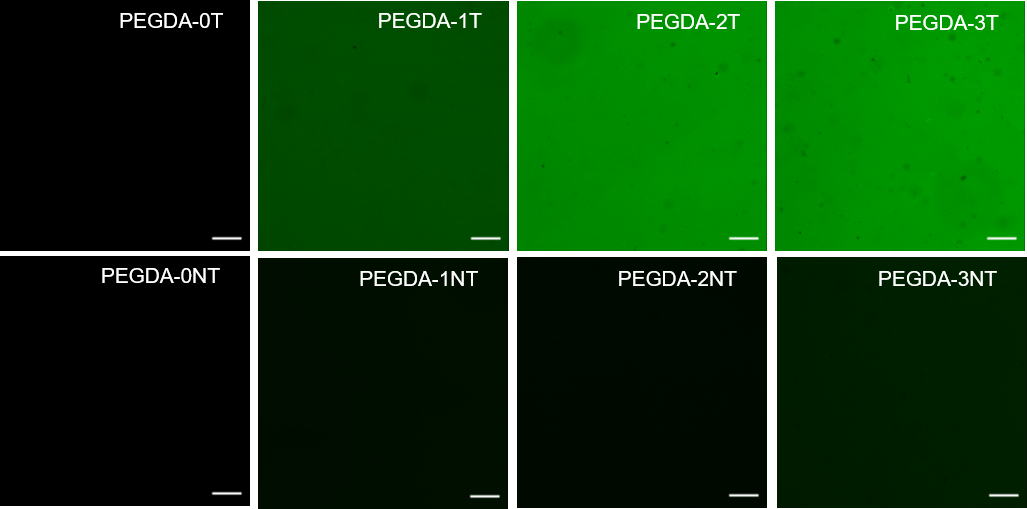


Figure SI 8. Maximum intensity projections of image stacks (z-stacks) recorded for fluorescence labelled hydrogels. Upper row: PEGDA-T hydrogels, samples treated with Na_2_S_2_O_5_. Lower row: PEGDA-NT hydrogels, controls in water. Scale bar 100 µm.


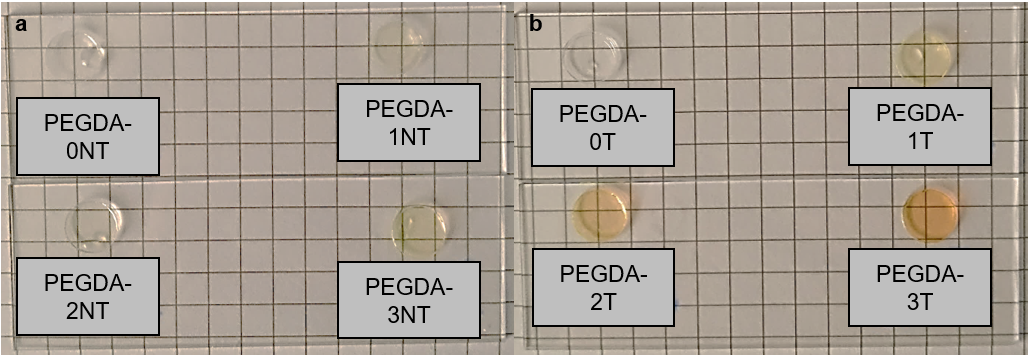


Figure SI 9. Photos of hydrogels after fluorescence staining. a) Non-treated PEGDA-NT hydrogels: PEGDA-0NT, PEGDA-1NT, PEGDA-2NT and PEGDA-3NT from upper left to lower right corner. b) Treated PEGDA-T hydrogels: PEGDA-0T, PEGDA-1T, PEGDA-2T and PEGDA-3T from upper left to lower right corner.

# Substrate conversion on isothiouronium-functional hydrogels





Figure SI 10. Absorption measurements of the ABTS substrate conversion on isothiouronium-functional hydrogels. Non-treated PEGDA-2NT hydrogels were agitated in a solution either with or without a maleimide-PEG_11_-biotin linker, and afterwards in a solution with streptavidin-coupled horseradish peroxidase.

1. Current address: Max Planck Institute for Intelligent Systems, Heisenbergstr. 3, 70569 Stuttgart, Germany. E-Mail: southan@is.mpg.de. [↑](#footnote-ref-1)
